# A Resource-Adaptive Routing Scheme with Wavelength Conflicts in Quantum Key Distribution Optical Networks

**DOI:** 10.3390/e25050732

**Published:** 2023-04-28

**Authors:** Tao Zhao, Xiaodong Fan, Bowen Dong, Quanhao Niu, Banghong Guo

**Affiliations:** Guangdong Provincial Key Laboratory of Nanophotonic Functional Materials and Devices, Guangdong Provincial Key Laboratory of Quantum Engineering and Quantum Materials, South China Normal University, Guangzhou 510006, China

**Keywords:** quantum key distribution, QKDON, RAWC, load balancing, wavelength conflicts

## Abstract

Quantum key distribution (QKD) has great potential in ensuring data security. Deploying QKD-related devices in existing optical fiber networks is a cost-effective way to practically implement QKD. However, QKD optical networks (QKDON) have a low quantum key generation rate and limited wavelength channels for data transmission. The simultaneous arrival of multiple QKD services may also lead to wavelength conflicts in QKDON. Therefore, we propose a resource-adaptive routing scheme (RAWC) with wavelength conflicts to achieve load balancing and efficient utilization of network resources. Focusing on the impact of link load and resource competition, this scheme dynamically adjusts the link weights and introduces the wavelength conflict degree. Simulation results indicate that the RAWC algorithm is an effective approach to solving the wavelength conflict problem. Compared with the benchmark algorithms, the RAWC algorithm can improve service request success rate (SR) by up to 30%.

## 1. Introduction

Optical fibers have been deployed worldwide [1] and serve as one of the most common inter-regional communication infrastructures. Due to the openness of the network and some vulnerabilities in existing communication technologies [2], optical networks are facing severe information security challenges. In recent years, malicious cyberattacks have occurred in many areas [3,4,5].

Classical encryption methods with mathematical complexity [6,7] have been quite challenged, especially in the face of quantum computing [8,9]. Most existing cryptographic systems are based on the Rivest–Shamir–Adleman (RSA) and elliptic-curve cryptography (ECC) algorithms [10]. However, the Shor quantum algorithm can crack the RSA and ECC algorithms in polynomial time [11]. Post-quantum cryptography (PQC) [12] and quantum key distribution (QKD) [13] can resist attacks from quantum computers. By using the PQC algorithm instead of the ECC or RSA algorithms, the security of today’s electronic commerce will be effectively guaranteed [14,15]. The PQC algorithm is executed efficiently on hardware systems [16]. It is usually subject to side-channel attacks during implementation, which affect security [17,18,19]. Lightweight PQC is typically implemented in a constrained environment with limited devices, where attackers access the device during normal device operation [20]. Thus, additional protection against electromagnetic attacks is required. Currently, the PQC algorithm is still in the stages of development and verification, and its application and popularization are facing many challenges. QKD can be implemented as it is a relatively mature encryption method against attacks.

QKD provides theoretically unconditional security when combined with the one-time pad encryption algorithm [21,22]. QKD is based on the basic law of quantum mechanics [23,24], so it is possible for both the sender and the receiver to detect any third-party intervention. To meet the requirements of secure multi-user communication, it is necessary to construct multi-point interconnected QKD networks. Because deploying dedicated optical fibers for QKD alone is too costly, we use wavelength division multiplexing (WDM) technology to multiplex quantum signals and classical signals into the same optical fiber for transmission [25]. It has been proven to be feasible to integrate QKD into optical networks by WDM technology [26,27,28,29]. In addition, to further improve the utilization of wavelength resources as well as the transmission capacity of the fiber, time division multiplexing (TDM) [30] and space division multiplexing (SDM) [31] can also be adopted in QKD optical networks (QKDON).

Software-defined networking (SDN) is a new type of network virtualization technique, whose core lies in the ability to define and control the networks programmatically [32]. The introduction of the SDN technique in optical networks can improve the programmability and flexibility of the networks. Recently, some researchers have proposed the architecture of QKD over software-defined optical networks [33]. In this network architecture, quantum key pools (QKPs) between two QKD nodes are constructed to store and manage quantum keys. The SDN controller is deployed to determine the configuration and employment of QKPs through the OpenFlow protocol. QKDON supporting SDN technique can efficiently manage the network-wide key resources in QKPs through centralized control.

Although the QKD technique is continuously being improved and the key generation rate is getting higher, it cannot fully meet the encryption needs of the explosive development of data services. Many different solutions are proposed to achieve resource allocation, including routing, wavelength, and quantum key methods. Table 1 compares focus points and performance metrics of the related works [33,34,35,36,37] with our scheme, where ‘-’ means that this issue is not involved in the corresponding paper. Ref. [33] proposed the KoD scheme to allocate resources and update keys for different services, considering key-updating cases based on time complexity and data complexity. The RWTA algorithm in ref. [34] assigned quantum key channels based on the key-updating periods to enhance security. The dynamic DSKP scheme was proposed in ref. [35] to generate and assign quantum keys for users’ different demands. Ref. [36] took the quantum key allocation process and the QKP key supplement process into account from the perspective of system efficiency and security, designing the DDKA scheme to satisfy the efficiency and lightweight requirements of IoT applications. To minimize the wastage of keys and maximize the utilization efficiency of resources, ref. [37] dynamically adjusted the link evaluation indicators by considering key volume, the key generation rate, and the number of path hops comprehensively, and selected the optimal path.

Most of the resource allocation algorithms in the aforementioned papers use the link length as the link weight, and carry out the routing allocation with the Dijkstra algorithm, while other schemes dynamically allocate resources for the security requirements of different service requests. Nevertheless, the quantum key generation rate is low, and the wavelength channels for data transmission are limited. We need to consider resource surplus status in QKDON. In addition, multiple service requests may arrive at the same time under practical application scenarios. These services compete for wavelength resources as they occupy the same link, resulting in service blocking.

This paper reconstructs the routing scheme and proposes a resource-adaptive algorithm with wavelength conflicts (RAWC). Firstly, the number of real-time network resources available is taken as an important factor in the scheme to achieve network load balancing. Then, we introduce the wavelength conflict mechanism to determine the communication path, so that the communication success rate is improved by minimizing wavelength competition.

The rest of this paper is organized as follows. Section 2 introduces the point-to-point QKD mechanism. Section 3 shows the QKD relay method used. Section 4 describes the SDN-based QKP technique. Section 5 presents the problems and application scenarios addressed by the scheme. Section 6 discusses the proposed RAWC scheme and algorithm. Section 7 shows and analyzes the simulation results. Section 8 discusses several issues that can be explored in future work. Finally, Section 9 concludes this paper.

## 2. Point-to-Point QKD Mechanism

This section describes the point-to-point QKD mechanism. BB84 protocol [13], the first QKD protocol, is the most technically mature protocol. In this protocol, the sender, Alice, encodes random binary bits using four single-photon polarization states, i.e., “horizontal (|H⟩)”, “vertical (|V⟩)”, “+45° (|+⟩)”, and “−45° (|−⟩)”, and sends them to the receiver, Bob, via the quantum channel (QCh). |H⟩ and |V⟩ constitute the Z basis, while |+⟩ and |−⟩ constitute the X basis, where the Z basis and the X basis are conjugate. Conventionally, suppose |H⟩ and |+⟩ represent the binary value 0, and |V⟩ and |−⟩ represent the binary value 1. 0 and 1 are called qubits. The correct bit information is obtained when Bob chooses the measurement basis corresponding to Alice to measure the received single photon. When Bob uses one measurement basis to measure the single-photon polarization state of another measurement basis, there is a 50% chance that he will receive different data from Alice.

**Figure 1:**
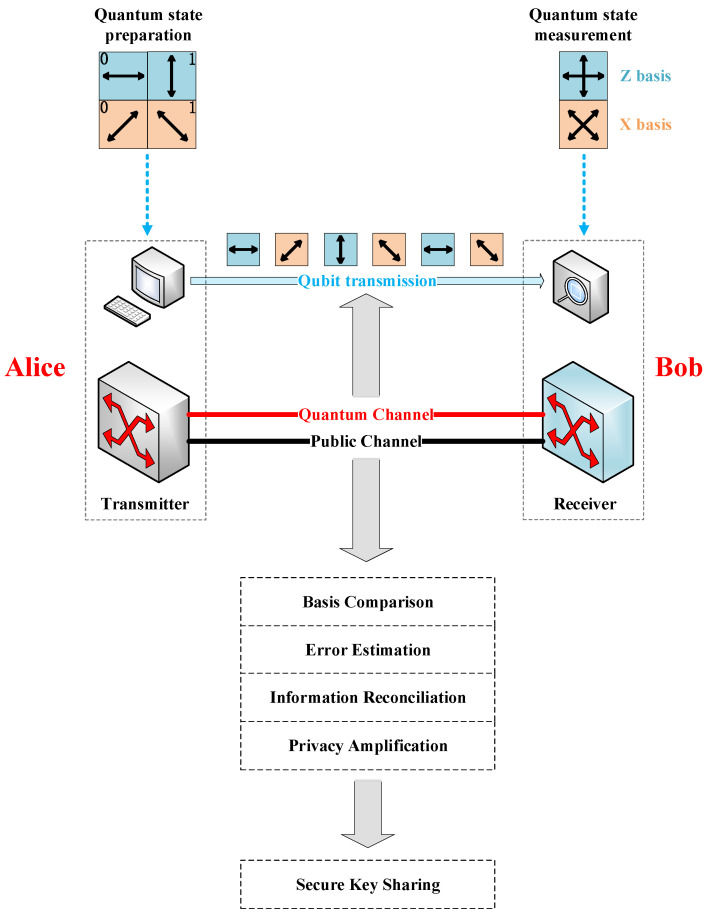
Point-to-point QKD mechanism based on BB84 protocol.

The point-to-point QKD mechanism based on the BB84 protocol is introduced below, as shown in Figure 1. The QKD process consists of the following steps:

1. Alice generates two random sequences of the same length, {a} and $\left\{a^{\prime }\right\}$, where {a} determines the chosen measurement basis and $\left\{a^{\prime }\right\}$ determines the single-photon polarization state to be sent. Alice prepares the single-photon polarization states (i.e., qubits, called raw keys) from the chosen random sequence and sends them to Bob via QCh.
2. Bob selects a random sequence {b} to determine the measurement basis, performs measurement for the received quantum states, and records the measurement results as $\left\{b^{\prime }\right\}$.
3. Alice announces the sequence {a} of the measurement basis chosen via the public channel (PCh). Bob compares it with his own sequence {b} of the measurement basis. When they choose the same basis, Alice and Bob keep the data and convert them into bit information (called sifted keys); otherwise, the data is discarded.
4. Alice and Bob disclose part of the sifted keys to perform error estimation and calculate the corresponding quantum bit error rate. If the quantum bit error rate is higher than a set error threshold, the QKD process is terminated, and step 1 is repeated.
5. Finally, Alice and Bob complete the post-processing process via PCh, including information reconciliation and privacy amplification, to obtain the final secure bits (called secret keys).

As shown in Figure 1, two channels are required for QKD, i.e., QCh for transmitting quantum signals and PCh for classical information exchange. To save fiber resources, we multiplex quantum signals and classical signals into the same optical fiber for transmission based on WDM technology. Given the crosstalk between classical and quantum signals, there is always a certain wavelength separation between QCh and PCh [28].

In recent years, researchers have paid more attention to the measurement-device-independent (MDI) protocol [38]. In the MDI protocol, the detection device is controlled by a third party, Charlie. Alice and Bob jointly send quantum signals to Charlie to complete the key sharing. This protocol eliminates the detector vulnerability of the actual QKD system. Since the proposal of the MDI protocol, research on long-distance and high-rate MDI-QKD in practical networks has increased [39,40], and its use has been extended to free space [41]. In addition, the multi-user scheme of the MDI protocol is useful for resisting detection attacks [42]. Similar to the MDI protocol, the twin-field (TF) protocol is also a three-party protocol. Based on single-photon interference, it can break through the rate–distance limit of QKD under the condition of guaranteeing the security of keys [43]. Minder et al. implemented a 454 km TF-QKD [44]. Wang et al. extended the security key distribution distance to 833.8 km [45].

## 3. Trusted Relay

QKDON is composed of multiple nodes and fiber links. However, the limited transmission distance of point-to-point QKD cannot accommodate QKD between two remote nodes. Hence, the distance of key distribution needs to be extended using relay technology [46]. In this paper, we use trusted relay-based networking technology.

**Figure 2:**
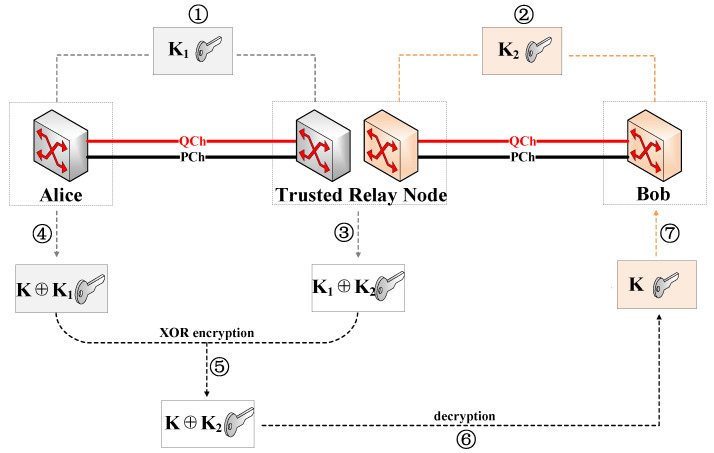
Schematic diagram of the relay principle.

As shown in Figure 2, assuming that Alice and Bob share a quantum key K via a trusted relay node, its relay principle can be summarized in the following steps: ➀ The shared key $K_{1}$ is generated between the sender Alice and the relay node in the form of QKD. ➁ The shared key $K_{2}$ is generated between the relay node and the receiver Bob. ➂ $\tilde{T}$he relay node performs XOR operation on key $K_{1}$ and key $K_{2}$ and stores the obtained key $K_{1}⊕K_{2}$ temporarily. ➃ Alice uses key $K_{1}$ to encrypt the key K to be shared with Bob and sends the obtained key $K⊕K_{1}$ to the relay node. ➄ The relay node performs an XOR operation with the temporarily stored key $K_{1}⊕K_{2}$ and key $K⊕K_{1}$ to obtain the encryption key $K⊕K_{2}$ and sends it to Bob. ➅ Bob decrypts the obtained key $K⊕K_{2}$ with the key $K_{2}$ to obtain key K. ➆ Finally, Alice and Bob obtain the shared key K.

The QKD path with multiple relay nodes has to be established for two end nodes with long distances. Then, the keys are transmitted along the QKD path using hop-by-hop encryption and decryption. As specified in the above steps, each relay node can obtain the key shared between two end nodes through decryption; hence, these relay nodes must be trusted. In QKDON, all QKD nodes can act as the source and destination nodes to request services and also as relay nodes to share quantum keys.

## 4. Quantum Key Storage and Provision

The key generation rate in the current QKD system is still low. Consequently, quantum keys are extremely valuable network resources in QKDON. Generally, a key storage device embedded in each QKD node is used to store quantum keys.

**Figure 3:**
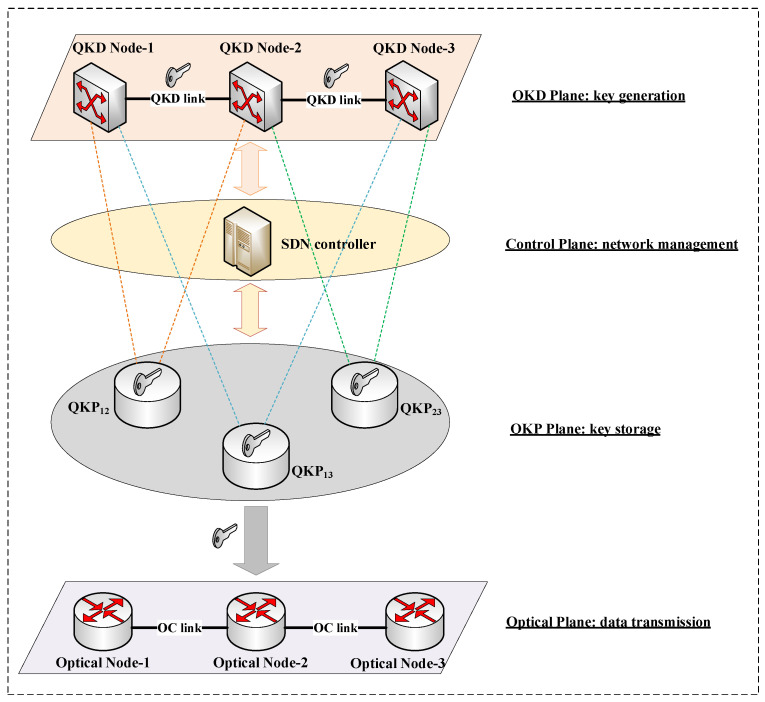
QKP architecture based on SDN.

Constructing a quantum key pool (QKP) can improve the quantum key provisioning capability [47]. We virtualize quantum keys generated between two QKD nodes and store them in a cache device, namely, QKP. Two QKD nodes directly connected via a fiber link can construct QKPs, such as ${\mathrm{QKP}}_{12}$ and ${\mathrm{QKP}}_{23}$ in Figure 3. Node-1 and Node-3 cannot distribute keys directly, so the end-to-end ${\mathrm{QKP}}_{13}$ is constructed using the relaying method mentioned in Section 3. QKP stores the generated keys continuously. Therefore, QKP supplies the accumulated keys in a timely manner when the encryption service is in high demand.

The key-sharing process between non-adjacent nodes requires quantum keys of each relay node. It is necessary to select the QKD path reasonably and manage the limited quantum key resources. QKP monitors the remaining number of quantum keys and the key generation rate in real time. The SDN controller manages QKPs through real-time information and schedules key resources to meet the encryption requirements [47]. The QKD links between QKD nodes in Figure 3 include QCh and PCh, which are used for the QKD process. Optical nodes are connected through optical communication links (OC links, i.e., data channels) to transmit service data. Based on WDM technology, the above three channels can be multiplexed into the same optical fiber [25].

## 5. Problem Statement

The wavelength channels for actual communication are selective. QCh and PCh for the QKD process and the data channel (DCh) for data transmission are usually placed in the C-band and O-band [25,48]. The total number of wavelengths should conform to the commercial WDM systems [34]. Additionally, the existing key generation rate only reaches the order of Mbps at the transmission distance of 50 km [49], so quantum keys in QKP are scarce resources. Therefore, it is important to allocate wavelengths and quantum keys efficiently and reasonably.

**Figure 4:**
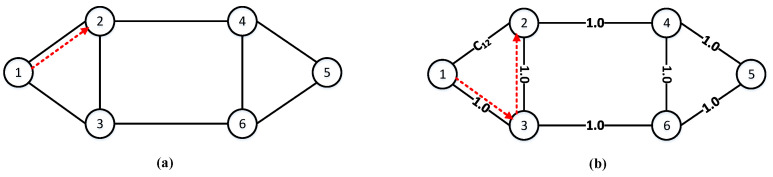
An example of routing assignment for the six-node network topology. (**a**) Static routing: fixed parameters such as link lengths are used as weights; (**b**) Dynamic routing: the real-time remaining number of resources is used as weights.

To clearly illustrate the above, the six-node network topology in Figure 4 is taken as an example. Now, we consider the change in the service request success rate and resource utilization. For simplicity, the node pair (1, 2) is used as the request node pair, where “1” and “2” represent the serial numbers of the source and destination nodes, respectively. As shown in Figure 4a, when only fixed parameters such as link lengths are used as link weights, the communication path selected by the Dijkstra algorithm is fixed as path 1→2 (red arrow). The continuous arrival of requests (1, 2) will aggravate the load on link 1→2, and may even run out of resources. Then, it is impossible to guarantee secure data transmission. Eventually, request (1, 2) is blocked. As shown in Figure 4b, when introducing the real-time residual wavelengths and quantum keys, the link weights dynamically change with the resource consumption of each service request. Assume that initially each link weight in Figure 4b is 1.0, and the weight of link 1→2 becomes $C_{12}$ at some point. If the relationship $C_{12}>C_{13}+C_{23}=1.0+1.0=2.0$ is satisfied, the shortest path for the request (1, 2) is path 1→3→2. Each time the request (1, 2) arrives, the communication path is selected according to the dynamically changing link weight. Thereafter, path 1→3→6→4→2 may also be taken as the communication path. In short-distance QKDON, where network resources are limited and some links do not carry services (such as link 1→3 and link 3→2, mentioned above), too many free wavelength channels and quantum keys will be wasted. If communication paths are selected based on the real-time remaining number of wavelengths available for each link, and the real-time remaining number of quantum keys available in QKP, the load pressure on the link is relieved. Finally, the encryption efficiency of the QKP is improved indirectly, and even the success rate of requests is increased.

**Figure 5:**
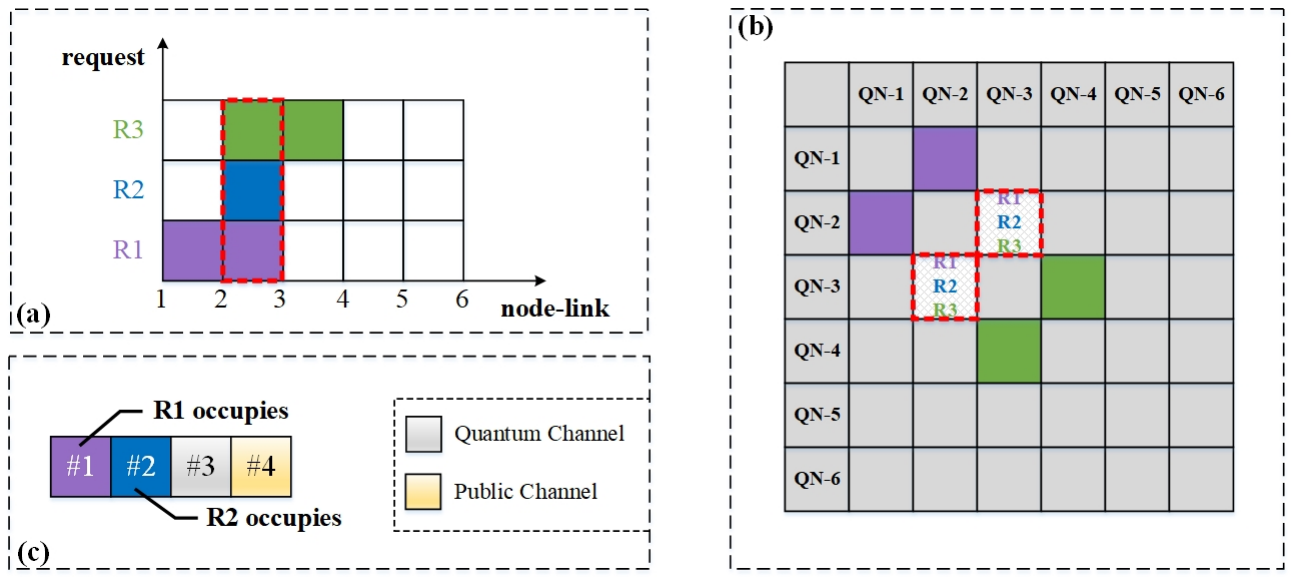
An example of wavelength conflicts. (**a**) Occupancy of links and wavelength channels by multiple services; (**b**) Mapping of resource occupancy in the adjacency matrix; (**c**) Wavelength occupancy for shared link 2→3.

Moreover, wavelength conflicts occur frequently when multiple service requests arrive simultaneously and occupy the same link, given the limited wavelength resources. Figure 5 demonstrates the potential conflicts due to multiple services sharing wavelength resources. As shown in Figure 5a, the horizontal coordinate represents the nodes and links, and the numbers on it represent the serial numbers of nodes. The colored boxes between every two numbers show the occupancy of the links and wavelength channels by different service requests and the vertical coordinate represents the service requests. Suppose three requests arrive simultaneously at a certain moment, namely requests R1: (1, 3), R2: (2, 3), and R3: (3, 4). Their communication paths are path 1→2→3, path 2→3, and path 3→2→4, respectively. It can be seen that the three requests share the wavelength resources on the link 2→3. Figure 5b shows the mapping of the corresponding case in Figure 5a in the adjacency matrix, where the upper and lower diagonal matrices are symmetrical, since the network topology is undirected. Figure 5c shows the wavelength channel occupancy of the shared link 2→3, with each box representing a wavelength channel on the link. For simplicity, consider the case where there are four wavelength channels on link 2→3. Channels #3 and #4 are used as QCh and PCh, respectively; the remaining two channels, #1 and #2, can only provide data transmission for two service requests. When R1, R2, and R3 arrive at the same time, we try to assign wavelength channels to them. At this time, two wavelength channels need to meet the data transmission requirements of three services; thus, wavelength conflicts will occur. Then, one service request must be blocked, such as the request R3, as shown in Figure 5c. Therefore, to improve the success rate of requests, we should avoid potential wavelength conflicts in the resource allocation process.

To summarize, which scheme to allocate limited wavelength resources and rare quantum keys to in order to reduce wavelength conflicts and blocking probability is an important research topic in short-distance QKDON.

## 6. The Proposed RAWC Scheme

### 6.1. Scheme Description

In this section, a resource-adaptive routing scheme with wavelength conflicts (RAWC) is proposed. The RAWC scheme is applied in scenarios where wavelength resources on links and quantum keys in QKP are limited and the simultaneous arrival of service requests occurs. Additionally, we consider short-distance QKDON, which means that point-to-point QKD can be carried out between any two nodes that are directly connected. The two nodes on the link generate keys directly via QCh and PCh, so the impact of link length on service requests can be ignored.

The RAWC scheme is divided into two specific cases from the perspective of routing allocation: ➀ Only one QKD service request arrives at a certain moment. Considering the characteristics of QKDON fully, the real-time remaining number of wavelengths available for each link and the real-time remaining number of quantum keys available in QKP are introduced into the calculation of link weights. After the arrival of each QKD service request, the communication path is calculated with the Dijkstra algorithm dynamically based on the resource surplus status. This facilitates the preference of the path with more wavelengths and quantum keys remaining to reduce the waste of resources on the service-free transmission link. In this way, network load balancing and the efficient utilization of network resources are achieved. ➁ Specifically, when *m*(m≥2) QKD service requests arrive at the same time, the K-shortest-path (KSP) algorithm is used to calculate the K shortest paths for each request. Then, the wavelength conflict degree of each of the K paths for the current request is calculated. The path with the lowest wavelength conflict degree is selected as the communication path, which is calculated according to the method described in Section 6.2.2. Considering the influence of other (m−1) requests, the path with low wavelength conflicts is preferentially selected when assigning routing for the current request. Therefore, the service blocking caused by potential wavelength competition will be reduced, which improves the request success rate. Table 2 lists some of the notations and definitions used in this paper.

### 6.2. Scheme Detail

In the following, we describe the steps and algorithm of the proposed RAWC scheme. Based on the analysis in Section 6.1, the path selection method for the RAWC scheme is described in detail below.

#### 6.2.1. Link Weight

The link weight is calculated according to the real-time remaining number of wavelengths available on each link and quantum keys available in the corresponding QKP:

(1) $$W\left(u,v\right)=\alpha \cdot \frac{\lambda_{ini}}{\lambda_{ava}\left(u,v\right)}+\beta \cdot \frac{K_{ini}}{K_{ava}\left(u,v\right)}$$

where W(u,v) denotes the weight of link(u,v); $\lambda_{ini}$ and $K_{ini}$ represent the initial number of wavelengths on each link and the initial number of quantum keys stored in QKP for each link, respectively, i.e., the maximum number of wavelengths and the maximum number of quantum keys; $\lambda_{ava}\left(u,v\right)$ and $K_{ava}\left(u,v\right)$ represent the real-time remaining number of wavelengths available on link(u,v) and the real-time remaining number of quantum keys available in QKP${}_{uv}$, respectively; and α and β are the weight adjustment factors (α+β=1). As can be seen in Equation (1), the more wavelength channels and quantum keys are available, the smaller the corresponding weight value.

When only one QKD service request arrives at a certain time, the Dijkstra algorithm is used to complete the routing calculation based on Equation (1). The path with the minimum weight value is taken as the communication path of the service.

#### 6.2.2. Wavelength Conflict Degree

When *m*(m≥2) QKD service requests (for ease of description, the m service requests are numbered $R_{1}$, $R_{2}$, …, $R_{m}$) arrive simultaneously at a given moment, wavelength conflicts caused by multiple services occupying the same link must be considered. The KSP algorithm is used to calculate K alternative paths for each service request. After determining the alternative paths for each service request, the wavelength conflict degree of each alternative path needs to be calculated. For service request $R_{i}\left(1\leq i\leq m\right)$, the wavelength conflict degree of its alternative path $p_{j}^{i}\left(1\leq j\leq K\right)$ is calculated as follows:

(2) $$\begin{array}{cc} \delta_{p_{j}^{i}} & =\sum_{\left(u,v\right)\in L_{p_{j}^{i}}}\left(\sum_{k=1(k\neq i)}^{m}\mu -\lambda_{ava}\left(u,v\right)\right)\\ \; & =\sum_{\left(u,v\right)\in L_{p_{j}^{i}}}\sum_{k=1(k\neq i)}^{m}\mu -\sum_{\left(u,v\right)\in L_{p_{j}^{i}}}\lambda_{ava}\left(u,v\right)\\ \; & =\sum_{\left(u,v\right)\in L_{p_{j}^{i}}}\sum_{k=1(k\neq i)}^{m}\mu -\lambda_{ava}\left(p_{j}^{i}\right) \end{array}$$

(3) $$\mu =\left\{\begin{array}{cc} 1, & \left(u,v\right)\in p^{k}\\ 0, & \left(u,v\right)\notin p^{k} \end{array}\right.$$

where $p_{j}^{i}$ denotes the *j*th (1≤j≤K) alternative path of the QKD service request $R_{i}$; $L_{p_{j}^{i}}$ is the link set contained in the path $p_{j}^{i}$; $\left(u,v\right)\in L_{p_{j}^{i}}$ is a link of this link set; $\lambda_{ava}\left(p_{j}^{i}\right)$ is the total number of available wavelengths for path $p_{j}^{i}$; $p^{k}$ represents the communication path selected for service request $R_{k}\left(1\leq k\leq m,k\neq i\right)$; and μ is the wavelength conflict marker of each link. When the communication path of request $R_{k}$ other than the service request $R_{i}$ contains link(u,v), it means that the two services have to share the wavelength resources of link(u,v); then, μ=1, otherwise μ=0. Note that the wavelength conflict degree is introduced to predict potential wavelength competition when choosing the path. It does not necessarily mean that wavelength conflicts will occur. The path with low wavelength conflicts is preferred to reduce the interference between service requests and improve the success rate of service requests.

The path selection procedure based on wavelength conflict degree is as follows:

1. Calculate the wavelength conflict degree of each alternative path for request $R_{1}$. Firstly, assume for the moment that the path $p_{1}^{k}\left(1<k\leq m\right)$ with the smallest weight of the other (m−1) service requests is their communication path, which is used to calculate the wavelength conflict degree of each alternative path for $R_{1}$. Then, the wavelength conflict degree $\delta_{p_{j}^{1}}$ of each alternative path $p_{j}^{1}\left(1\leq j\leq K\right)$ for $R_{1}$ is calculated according to Equation (2). Finally, the path with the minimum wavelength conflict degree is selected as the communication path, denoted as $p^{1}$.
2. Calculate the wavelength conflict degree of each alternative path for request $R_{2}$. According to step 1, when calculating the wavelength conflict degree of the alternative paths for $R_{2}$, the communication path of $R_{1}$ is determined as $p^{1}$. The path $p_{1}^{k}\left(2<k\leq m\right)$ with the smallest weight of the other (m−2) service requests is assumed to be their communication path. Then, calculate the wavelength conflict degree $\delta_{p_{j}^{2}}$ of each alternative path $p_{j}^{2}\left(1\leq j\leq K\right)$ for $R_{2}$. Finally, select the path with the smallest wavelength conflict degree as the communication path, denoted as $p^{2}$.
3. Calculate the wavelength conflict degree of each alternative path for request $R_{3}$. According to steps 1 and 2, the communication paths for $R_{1}$ and $R_{2}$ are $p^{1}$ and $p^{2}$, respectively. Assume that the path $p_{1}^{k}\left(3<k\leq m\right)$ with the smallest weight of the other (m−3) service requests is their communication path. Then, calculate the wavelength conflict degree $\delta_{p_{j}^{3}}$ of each alternative path $p_{j}^{3}\left(1\leq j\leq K\right)$ for $R_{3}$. Finally, select the path with the smallest wavelength conflict degree as the communication path, denoted as $p^{3}$.

And so on and so forth, until the communication paths of all service requests are calculated.

### 6.3. RAWC Algorithm

Based on the above scheme description, the RAWC algorithm is designed. It is divided into two cases: ➀ Only one QKD service request arrives at a certain time, in which case there is no wavelength conflict, as shown in Algorithm 1; ➁ Multiple QKD service requests arrive simultaneously at a certain time and there are wavelength conflicts, as shown in Algorithm 2. The schemes for both cases are divided into three steps, i.e., routing calculation, wavelength assignment, and quantum key assignment. In the routing computation, case 1 needs to adaptively calculate the communication path with the Dijkstra algorithm for the QKD service request arriving at each moment. Case 2 first uses the KSP algorithm to calculate K alternative paths for each QKD service request, and then calculates the wavelength conflict degree of each alternative path.
**Algorithm 1** The case without wavelength conflicts**Input:** G(V,E), $R_{i}\left(s_{R_{i}},\;d_{R_{i}},\;K_{R_{i}}\right)$, $\lambda_{ini}$, $K_{ini}$, α, β **Output:** SR, WRU, QKU of QKD service requests
1:**for** each service request $R_{i}$ **do**2:    routing computation with the Dijkstra algorithm according to Equation (1);3:    search available wavelength resources $W(p_{R_{i}})$ through the selected path $p_{R_{i}}$;4:    **if** $W\left(p_{R_{i}}\right)\neq ⌀$ **then**5:        select an available wavelength with the first-fit (FF) algorithm;6:        update each link wavelength resource status of path $p_{R_{i}}$;7:    **else**8:        the service request is blocked;9:        **continue**;10:    **end if**11:    **for** each link(u,v) of path $p_{R_{i}}$ **do**12:        quantum key assignment;13:        **if** $K_{R_{i}}\leq K_{ava}\left(u,v\right)$ **then**14:           select $K_{R_{i}}$ quantum keys from QKP for $R_{i}$ with the FF algorithm;15:           update the remaining quantum key status of each node of path $p_{R_{i}}$;16:        **else**17:           the service is insecure and blocked;18:        **end if**19:    **end for**20:**end for**


**Algorithm 2** The case with wavelength conflicts**Input:** G(V,E), $R_{i}\left(s_{R_{i}},\;d_{R_{i}},\;K_{R_{i}}\right)$, $\lambda_{ini}$, $K_{ini}$, α, β **Output:** SR, WRU, QKU of QKD service requests
1:**for** simultaneous *m*(m≥2) service requests $R_{1}$, $R_{2}$, …, $R_{m}$ **do**2:    path selection for each service request based on the wavelength conflict degree;3:    **for** each service request $R_{i}\left(1\leq i\leq m\right)$ **do**4:        routing computation with the KSP algorithm according to Equation (1);5:        **for** each computed alternative path $p_{j}^{i}\left(1\leq j\leq K\right)$ **do**6:           wavelength conflict computation referring to Section 6.2.2;7:        **end for**8:        select the path with the least wavelength conflict as the communication path $p^{i}$;9:        search available wavelength resources $W(p^{i})$ through the path $p^{i}$;10:        **if** $W(p^{i})\neq ⌀$ **then**11:           select an available wavelength with the FF algorithm;12:           update each link wavelength resource status of path $p^{i}$;13:        **else**14:           the service request is blocked;15:           **continue**;16:        **end if**17:        **for** each link(u,v) of path $p^{i}$ **do**18:           quantum key assignment;19:           **if** $K_{R_{i}}\leq K_{ava}\left(u,v\right)$ **then**20:               select $K_{R_{i}}$ quantum keys from QKP for $R_{i}$ with the FF algorithm;21:               update the remaining quantum key status of each node of path $p^{i}$;22:           **else**23:               the service is insecure and blocked;24:           **end if**25:        **end for**26:    **end for**27:**end for**


## 7. Simulation Results and Analysis

To evaluate the feasibility and performance of the RAWC algorithm in QKDON, the simulation was performed on both the six-node network topology (shown in Figure 5) and the NSFNET topology, which consists of 14 nodes and 21 links. This paper studies short-distance QKDON with limited network resources and scenarios where multiple QKD service requests arrive simultaneously. In our work, the quantum channel and the public channel occupy two wavelengths, and each link has the same total number of wavelengths for the data channel. Without special instructions, there are 40 wavelengths on each link. In addition, it is assumed that each QKP stores the same number of quantum keys. In the following simulation, we set the link weighting factors to α=0.5,β=0.5. The arrival of QKD service requests follows a Poisson distribution. The holding time of each service request is fixed and the same. When the duration of the services at a certain moment is over, the services at the next moment arrive. The channels are released when the services end their occupation of the data channels. To select more alternate paths, Section 6.2.2 employs the KSP algorithm with K=3.

This section evaluates the performance of this scheme by comparing different schemes based on the following three metrics: service request success rate (SR), wavelength resource utilization (WRU), and quantum key utilization (QKU). SR refers to the proportion of QKD services that can successfully obtain quantum keys from QKP and can be successfully transmitted to the total services, indirectly reflecting the encryption efficiency of QKP and the ability of the network to guarantee service transmission. WRU and QKU are utilized to describe the usage of network resources of QKDON.

### 7.1. Verification of the RAWC Algorithm on the Six-Node Network Topology

**Figure 6:**
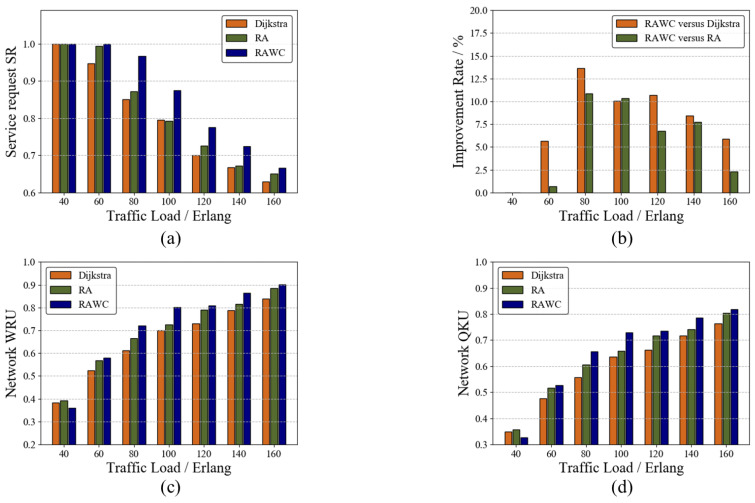
Performance comparison of different algorithms on the six-node network topology. (**a**) SR; (**b**) Improvement rate; (**c**) WRU; (**d**) QKU.

Figure 6 shows the performance of this scheme on the six-node network topology. The Dijkstra routing algorithm and the resource-adaptive (RA) algorithm are used as two benchmark schemes to verify the performance improvement in the RAWC algorithm in terms of the service request success rate and resource utilization. In this paper, the Dijkstra routing algorithm takes the link length as the link weight, and the RA algorithm does not consider wavelength conflicts. Figure 6a,b compare SRs of different algorithms, and the percentage improvement in the RAWC algorithm in SR compared with other algorithms, respectively. As the traffic load increases, the SRs of all three algorithms decrease. Due to limited network resources, the increase in traffic load reduces the ability of QKDON to provide wavelength channels and QKP to provide key services. Additionally, it can be observed that the lowest SR is achieved under the Dijkstra algorithm, and the highest SR under the RAWC algorithm. At a traffic load of 80 Erlang, the proposed RAWC algorithm improves SR by up to 13.5% compared with the Dijkstra algorithm. This is because using only the path length as the link weight increases the link load pressure, which causes service blocking. Even if a dynamic routing scheme is adopted, not taking into account the competition for wavelength resources blocks services. Figure 6c,d show the wavelength resource utilization (WRU) and quantum key utilization (QKU) of the three schemes, respectively. From the figures, it can be seen that both WRU and QKU increase with the increase in traffic load, which is the result of increased network resource consumption. When the traffic load is 40 Erlang, the advantages of the RAWC algorithm are not observable because the traffic is low. As the traffic load increases gradually, WRU and QKU of the proposed RAWC algorithm are consistently found to be the highest. The RAWC algorithm gives priority to the links with more remaining resources when selecting the communication path. The above simulation results show that the RAWC algorithm is the optimal choice for achieving load balancing.

### 7.2. Service Request SR on Two Network Topologies

#### 7.2.1. Comparison of Different Schemes

**Figure 7:**
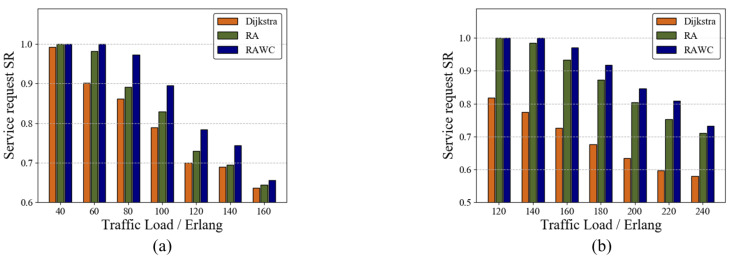
SR of the three schemes. (**a**) Six-node network topology; (**b**) NSFNET topology.

To further verify the performance of the RAWC scheme, we applied the algorithm on both the six-node topology and the NSFNET topology. Figure 7a,b illustrate the results of SR versus traffic load for the three schemes on the six-node network topology and the NSFNET topology, respectively, with other initial conditions consistent in both networks. The SR decreases as the traffic load increases in both networks, with the RAWC scheme always having a higher SR than the other two schemes. On NSFNET topology, the proposed RAWC scheme improves SR by 30% on average compared with the Dijkstra algorithm. When the traffic load increases to a certain level, the gap between the RA scheme and the RAWC scheme gradually decreases. The reason is that the moment of arrival of multi-service requests set in this paper is fixed. As the service traffic base increases, the proportion of service requests completed by the RAWC scheme compared to the RA scheme decreases, which will be reflected in Section 7.2.3.

#### 7.2.2. Service Request SR of the RAWC Scheme in Different Resource Cases

**Figure 8:**
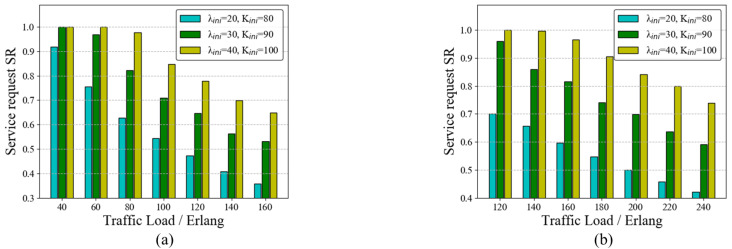
SR of the RAWC algorithm in different cases (case 1: $\lambda_{ini}=20,K_{ini}=80$; case 2: $\lambda_{ini}=30,$$K_{ini}=90$; case 3: $\lambda_{ini}=40,K_{ini}=100$). (**a**) Six-node network topology; (**b**) NSFNET topology.

In this paper, we consider the following three resource cases: ➀ Case 1: the initial number of wavelength channels $\lambda_{ini}$ in the network is set to 20, and the initial number of quantum keys $K_{ini}$ stored in QKP corresponding to each link is set to 80; ➁ Case 2: $\lambda_{ini}=30,K_{ini}=90$; ➂ Case 3: $\lambda_{ini}=40,K_{ini}=100$. The SRs of the RAWC scheme in the three cases on the six-node network topology and the NSFNET topology are shown in Figure 8a,b, respectively. It is clear that with a fixed traffic load, the more initial resources are in QKDON, the higher the service request success rate is. This indicates that initial resources in the network should be supplemented as much as possible for QKP to ensure more secure service transmission.

#### 7.2.3. Service Request SR of the RAWC Scheme under Different Scenarios

**Figure 9:**
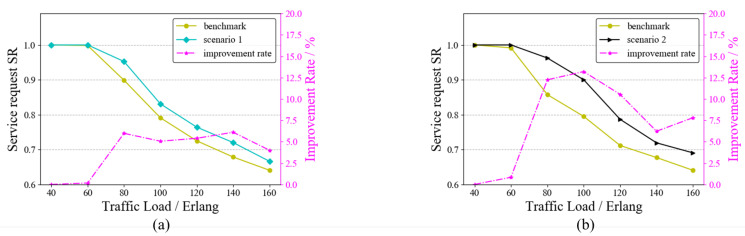
SR of the RAWC algorithm under different scenarios on the six-node network topology. (**a**) Scenario 1; (**b**) Scenario 2.

**Figure 10:**
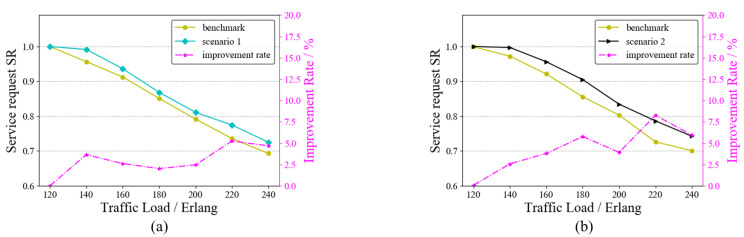
SR of the RAWC algorithm under different scenarios on NSFNET topology. (**a**) Scenario 1; (**b**) Scenario 2.

In the service request model of this paper, there may be one or more services arriving at each moment. Multiple requests arriving at one moment may lead to wavelength conflicts. To test the ability of the RAWC algorithm to solve the wavelength conflict problem, we set up two scenarios: ➀ Scenario 1: there are four moments with multiple service requests arriving at the same time; ➁ Scenario 2: there are eight moments with multiple service requests arriving at the same time. As a comparison, both the benchmark algorithm and the RAWC algorithm were applied to these two scenarios, where the benchmark algorithm is RA and no competition for wavelength resources is considered. Figure 9 and Figure 10 demonstrate the results of the two algorithms on the six-node network topology and the NSFNET topology, respectively. The vertical coordinates on the right side indicate the percentage improvement in the RAWC algorithm in SR compared with the benchmark scheme under each scenario. As shown in Figure 9a,b, the RAWC algorithm (cyan line in Figure 9a and black line in Figure 9b) has a higher SR than the benchmark algorithm (yellow line in all Figures) under both scenario 1 and scenario 2. In addition, the RAWC algorithm under scenario 2 has a higher improvement over the benchmark algorithm for the same traffic load compared to scenario 1. Under scenario 1, the improvement rate is up to 6% at a traffic load of 140 Erlang; under scenario 2, the improvement rate is up to 13% at a traffic load of 100 Erlang. The reason is that there is a higher number of multiple services under scenario 2, and the RAWC algorithm can better avoid the path with more wavelength conflicts to meet the transmission requirements of more services than the benchmark algorithm. Figure 10a,b show the comparison results of the two schemes on NSFNET under scenario 1 and scenario 2, respectively. It should be noted that the simulation results on NSFNET topology are similar to the six-node network topology, except for some differences in performance improvement. On NSFNET topology, the maximum SR improvement in the RAWC scheme is no more than 10%, which is related to the network topology itself. To summarize, for the problem of multi-service request arrival, we can effectively improve the network performance by considering wavelength conflicts in the dynamic routing scheme.

## 8. Open Discussion and Future Outlook

In this work, we attempted to investigate the resource conflict problem in the practical application scenario of QKDON and present a model to solve the problem. Our results validate the feasibility of the model. Nevertheless, some assumptions are made in this model, such as the initial number of wavelengths and keys and the service traffic. We aim to consider the impact of QKD on the network comprehensively. We discuss several issues which will be explored in future work, below.

1. **Allocation of multiple network resources:** The routing scheme proposed in this paper only considers the allocation of the wavelength and key resources. Based on the use of WDM technology to construct QKDON, TDM and SDM technologies further improve resource utilization and transmission capacity. Therefore, the introduction of the above two techniques and the allocation of time slots and fiber resources can be considered in future research.
2. **Survivability of QKDON:** With the development of QKD technology, the survivability issues of QKDON have also received extensive attention. Actually, due to factors such as natural disasters or man-made damage, link failure will inevitably occur. The study of the survivability of QKDON is of great importance for the practicality of QKD. Survivability involves two aspects: protection and recovery. Different from classical optical networks, the failure of QKDON will make it impossible to generate quantum keys. Therefore, how to protect the quantum channel in the network and recover the service transmission in time after the failure is an important research topic.
3. **Applicability of QKD and other encryption methods:** Post-quantum cryptography (PQC) is another secure encryption method that can be used to resist quantum computing in addition to QKD. QKD solves the key distribution problem, while PQC studies the security of cryptographic algorithms in quantum environments. QKD and PQC differ in terms of practical conditions and application scenarios. Assessing the applicability of QKD and PQC for different security requirements is a worthwhile research topic in the network. In addition, we need to compare the costs of deploying QKD devices and PQC in different security-demanding circumstances.

## 9. Conclusions

In this paper, a routing scheme for QKD optical networks (QKDON) is described. In QKDON with limited resources, multiple services arriving at the same time may lead to wavelength conflicts. By introducing the wavelength conflict mechanism and considering the change in network resources, the RAWC routing scheme is presented to assign keys and wavelengths and select the communication path with the least conflict. The performance of the RAWC algorithm is evaluated under different scenarios on the six-node network topology and the NSFNET topology. Simulation results show that the RAWC algorithm has a higher service request success rate and resource utilization than the benchmark scheme. This means that our scheme is beneficial for load balancing and effectively solves the problem of wavelength competition. In addition, increasing initial resources in the network can ensure the secure transmission of more services. In future work, the allocation of multiple network resources and multiple service requirements will be further investigated.

## Figures and Tables

**Table 1:** The comparison of the related schemes with the RAWC scheme.

Schemes	Focus Points	Security Probability/ Success Rate	Resource Utilization	Improvement Rate to Benchmark
KoD [33]	security requirements of different services	up to 1.0	-	-
RWTA [34]	static routing, wavelength, and time-slot assignment	up to 1.0	wavelength utilization: up to 0.5; time-slot utilization: up to 0.75	time-slot utilization: 0.16
DSKP [35]	the number of keys in QKP	-	time-slot utilization: up to 0.55	-
DDKA [36]	requirements of the IoT application	up to 0.95	-	SR: up to 0.26
ADA [37]	key volume, key update rate and path hops required by the application	up to 1.0	-	SR: 0.11 on average
RAWC (our scheme)	resource status of the network and wavelength conflicts	up to 1.0	wavelength utilization: up to 0.9	SR: up to 0.3

**Table 2:** Notations and definitions.

Notations	Definitions
G(V,E)	QKDON network topology
*V*	the node sets of QKDON
*E*	the bi-directional fiber link sets of QKDON
W(u,v)	the weight of link(u,v)
QKP${}_{uv}$	QKP corresponding to link(u,v)
$\lambda_{ini}$	the initial number of wavelengths on each link
$K_{ini}$	the initial number of quantum keys stored in QKP for each link
$\lambda_{ava}\left(u,v\right)$	the real-time remaining number of wavelengths available on link(u,v)
$K_{ava}\left(u,v\right)$	the real-time remaining number of quantum keys available in QKP${}_{uv}$
$R_{i}\left(s_{R_{i}},\;d_{R_{i}},\;K_{R_{i}}\right)$	the QKD service request
$s_{R_{i}}$	the source node of the QKD service request $R_{i}$
$d_{R_{i}}$	the destination node of the QKD service request $R_{i}$
$K_{R_{i}}$	the number of quantum keys required for the QKD service request $R_{i}$
$p_{j}^{i}$	the *j*th alternative path of the QKD service request $R_{i}$
$\delta_{p_{j}^{i}}$	the wavelength conflict degree of the *j*th alternative path for the QKD service request $R_{i}$
$\lambda_{ava}\left(p_{j}^{i}\right)$	the total number of available wavelengths for path $p_{j}^{i}$
μ	wavelength conflict marker
$p_{R_{i}}/p^{i}$	the communication paths of the QKD service request $R_{i}$ in two cases
SR	the success rate of QKD service requests
WRU	wavelength resource utilization
QKU	quantum key utilization

## Data Availability

Not applicable.
